# Meta-analysis of transcatheter arterial chemoembolization plus radiofrequency ablation versus transcatheter arterial chemoembolization alone for hepatocellular carcinoma

**DOI:** 10.18632/oncotarget.13813

**Published:** 2016-12-07

**Authors:** De-jun Yang, Kun-lun Luo, Hong Liu, Bing Cai, Guo-qing Tao, Xiao-fang Su, Xiao-juan Hou, Fei Ye, Xiang-yong Li, Zhi-qiang Tian

**Affiliations:** ^1^ Department of Gastrointestinal Surgery, The Changzheng Hospital, Second Military Medical University, Shanghai, 200003, China; ^2^ Department of General Surgery, Wuxi People's Hospital Affiliated Nanjing Medical University, Wuxi 214023, China; ^3^ Department of General Surgery and Rehabilitation Medicine and Oncology, The 101st Hospital of Chinese PLA, Wuxi 214044, China; ^4^ Tumor Immunology and Gene Therapy Center, Eastern Hepatobiliary Surgery Hospital, The Second Military Medical University, Shanghai 200438, China

**Keywords:** transcatheter arterial chemoembolization, radiofrequency ablation, hepatocellular carcinoma, meta-analysis

## Abstract

This meta-analysis was conducted to compare transcatheter arterial chemoembolization (TACE) plus radiofrequency ablation (RFA) with TACE alone for hepatocellular carcinoma. We searched MEDLINE, EMBASE and CENTRAL for all relative randomized controlled trials (RCTs) and retrospective studies until October 31 2016. Tumor response, recurrence-free survival, overall survival and postoperative complications were the major evaluation indices. Review Manager (version 5.3) was used to analyze the data. Dichotomous data was calculated by odds ratio (OR) with 95% confidence intervals (CI). There were 1 RCT and 10 retrospective studies with 928 patients in this meta-analysis: 412 patients with TACE plus RFA and 516 patients with TACE alone. Compared with TACE alone group, TACE plus RFA group attained higher tumor response rates (OR = 6.08, 95% CI = 4.00 to 9.26, *P* < 0.00001), achieved longer recurrence-free survival rates (OR_RFS_ = 3.78, 95% CI: 2.38 to 6.02, *P* < 0.00001) and overall survival rates (OR_1-year_ = 3.92, 95% CI = 2.41–6.39, *P* < 0.00001; OR_3-year_ = 2.56; 95% CI = 1.81–3.60; *P* < 0.00001; OR_5-year_ = 2.78; 95% CI = 1.77–4.38; *P* < 0.0001). Serious postoperative complications were not observed, although complications were higher in TACE plus RFA group than that in TACE alone group (OR = 2.74, 95% CI = 1.07 to 7.07, *P* = 0.04). In conclusion, the use of TACE plus RFA for intermediate stage hepatocellular carcinoma can attain higher tumor response rates and improve survival rates than TACE alone.

## INTRODUCTION

Hepatocellular carcinoma has high malignant degree and causes nearly one million deaths each year worldwide [1, 2]. Hepatocellular carcinoma frequently occurs in the setting of cirrhosis [4], because hepatocellular carcinoma carcinogenesis was usually associated with hepatitis virus and chronic alcoholism [3]. The tumor mass and the patient's hepatic reserve are most important in determining a patient's prognosis and potential treatment options. Surgical therapies are mainly treatment options for hepatocellular carcinoma that offer the chance of potential cure, either by orthotopic liver transplantation or hepatic resection [5, 6]. Unfortunately, only a portion of hepatocellular carcinoma patients are suitable for surgical therapies because of unfavorable location, the presence of multiple tumors, poor hepatic reserve and shortage of donor livers [7].

At present, transarterial chemoembolization (TACE), radiofrequency ablation (RFA), radiotherapy, radioembolization, and percutaneous ethanol injection (PEI) are mainly non-surgical approaches for unresectable hepatocellular carcinoma patients [2, 8]. The early stage hepatocellular carcinoma is treated with RFA and PEI, but intermediate stage and large unresectable hepatocellular carcinoma are tend treated with TACE according to Barcelona Clinic for Liver Cancer (BCLC) system [9, 10]. However, TACE alone is very hard to achieve complete necrosis of liver tumor and the long term prognosis is unsatisfactory [11]. Combining TACE and RFA for treatment hepatocellular carcinoma can theoretically have a synergistic effect. The use of TACE plus RFA of treatment for small hepatocellular carcinoma is a common practice [12]. However, it is still controversial whether the effect of TACE plus RFA for intermediate stage hepatocellular carcinoma is better than that of TACE alone [13, 14].

A meta-analysis will be helpful to attain definitive proof from all of the available studies. Therefore, this meta-analysis was conducted to compare TACE plus RFA with TACE alone for intermediate stage hepatocellular carcinoma.

## RESULTS

### Search results and quality assessment

**Figure 1:**
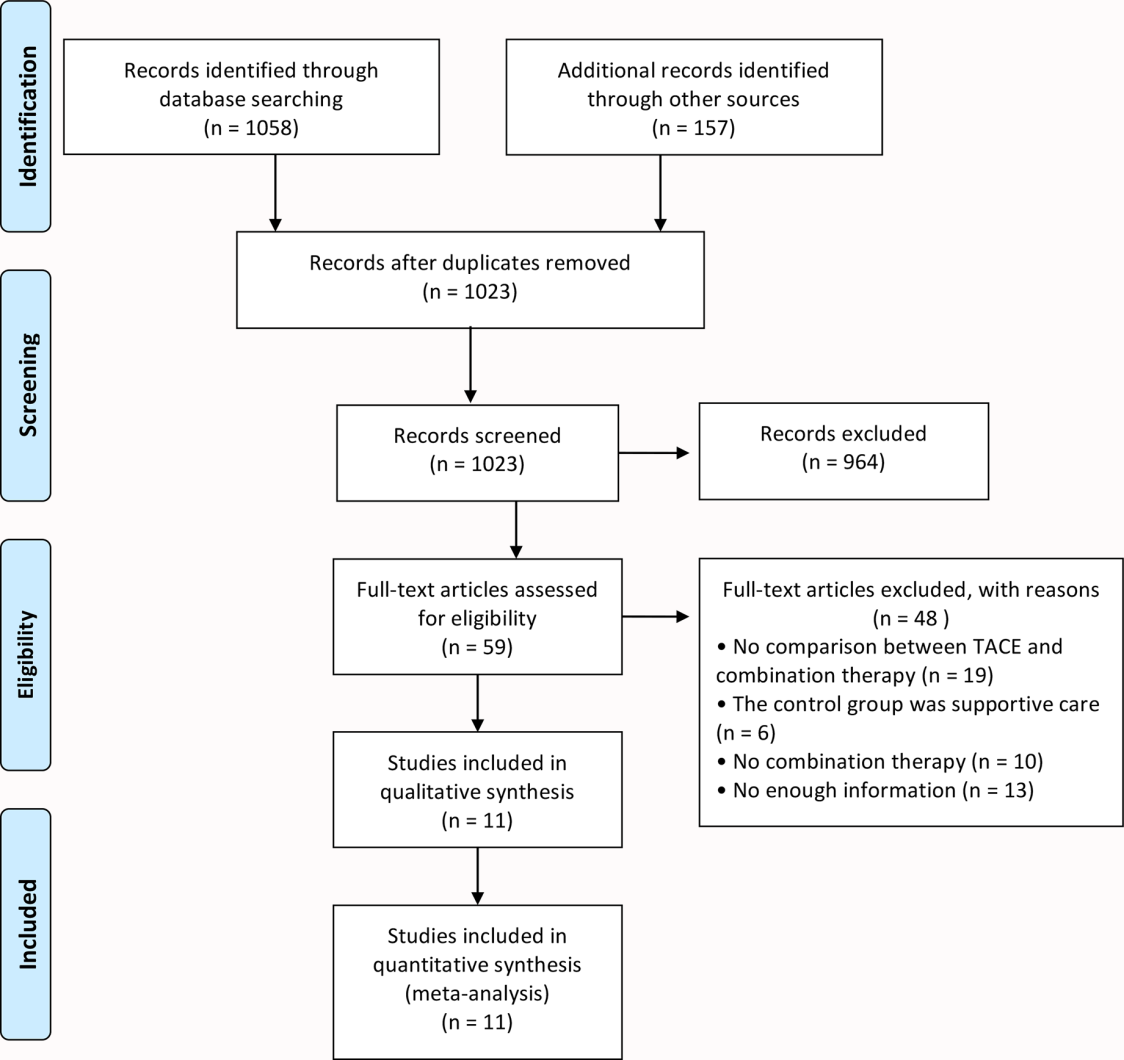
Flow diagram showing the detailed selection process of this meta-analysis

There were 1023 studies identified by a combined search of electronic databases (MEDLINE, EMBASE, and CENTRAL) and a manual approach until October 31th 2016. After title and abstract of the studies were identified, 964 studies were excluded accorded with the research criteria. The remaining 59 studies were subjected to a full-text review. And then, an additional 48 articles were excluded for the reasons described in Figure 1. In the end, this meta-analysis included a total of 11 studies: 1 randomized controlled trial [15], 10 retrospective studies [16–25]. Figure 1 showed the detailed selection process diagram.

**Figure 2:**
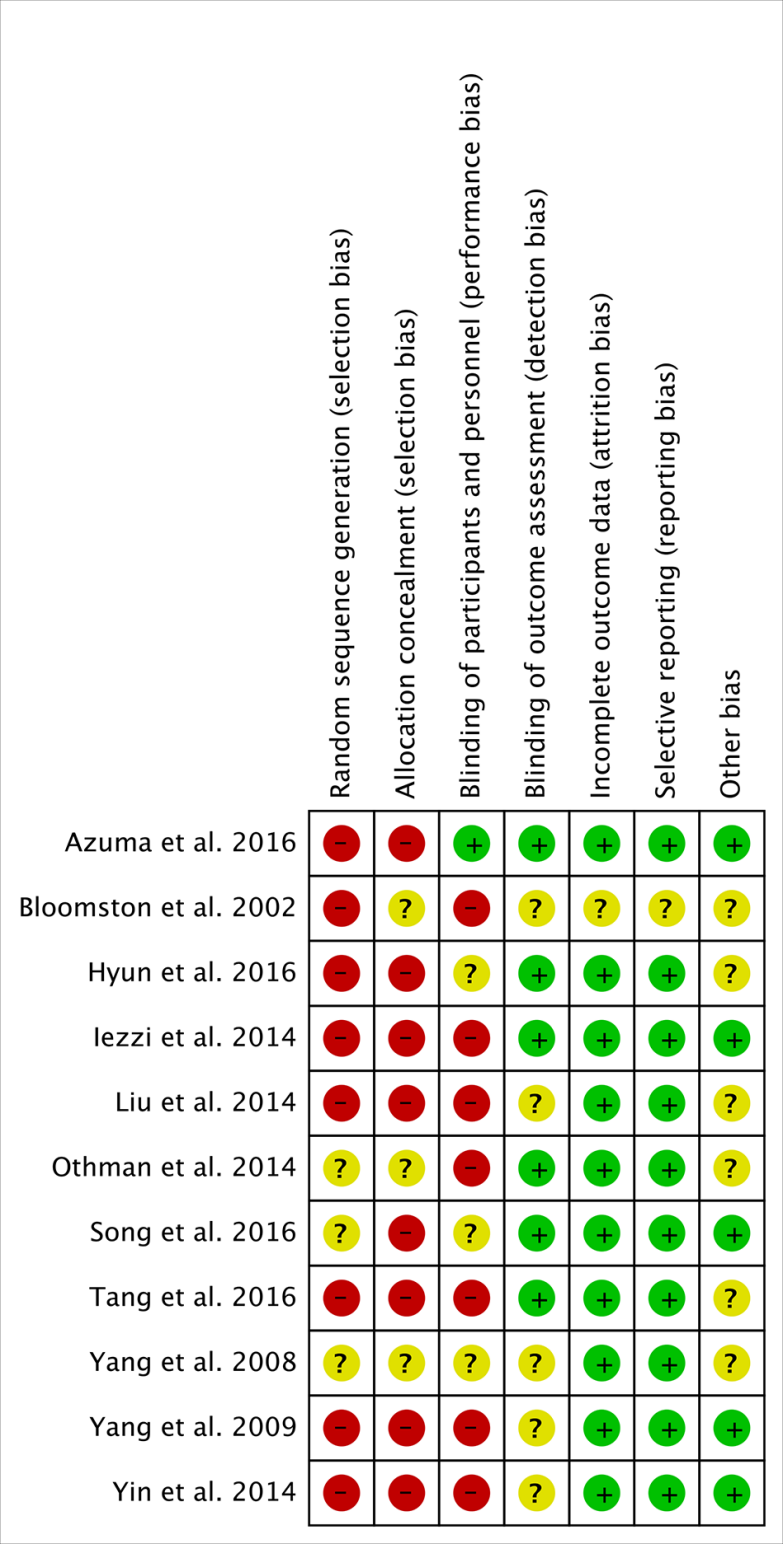
The summary of risk of bias for each study

**Figure 3:**
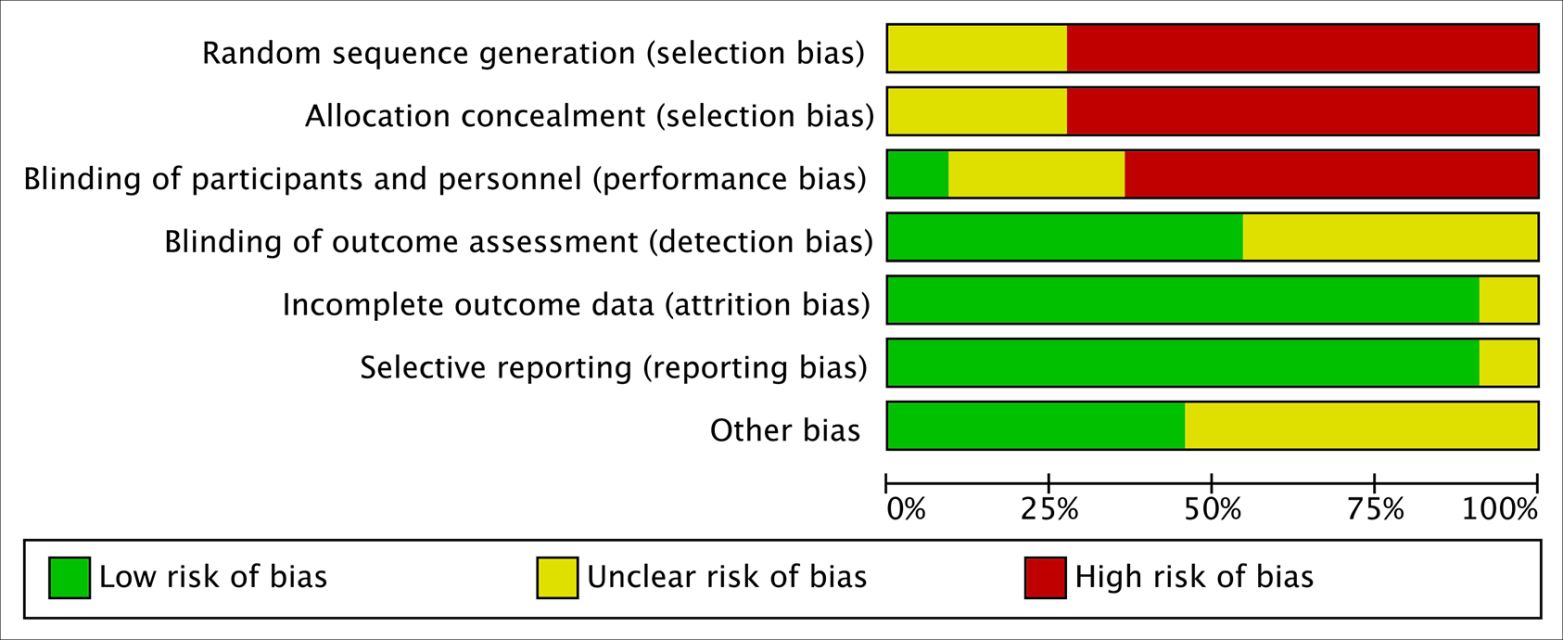
The bar charts as percentages showing the risk of bias of each item in all included studies.

A total of 928 hepatocellular carcinoma patients were included in the meta-analysis: 412 patients with TACE plus RFA and 516 patients with TACE alone. Table 1 summarized the description of patients at baseline from the studies of this meta-analysis. Risk of bias was assessed using the criteria of Cochrane Collaboration's tool [26] (Figure 2). High risk of selection bias and performance bias were more than 50 percent, but risk of detection, attrition, reporting and other biases were not apparent. Therefore, this meta-analysis has a high risk of selection bias (Figure 3).

**Table 1:** Basic clinical characteristics of the included studies in this meta-analysis

Studies (Author, year, country)	Design	Treatment	No. of patients	Age (years)	Sex (M/F)	Tumor size (cm)	Child-Pugh Class (A/B/C)	No. of tumors (1 /≥ 2)	Mean follow up (months)
Azuma *et al.*, 2016 (Japan)	Non-RCT	TACE + RFA TACE	20 39	69 (52–82) 70 (48–92)	14/6 25/14	1.3 (0.4–5.0) 1.3 (0.4–5.2)	16/4/0 28/11/0	0/20 0/39	NA NA
Bloomston *et al.*, 2002 (American)	Non-RCT	TACE + RFA TACE	13 24	61.1 ± 9.4 64.3 ± 11.9	12/1 15/9	NA NA	NA NA	NA NA	9.1 ± 7.1 9.1 ± 7.1
Hyun *et al.*, 2016 (Korea)	Non-RCT	TACE + RFA TACE	37 54	57.7 ± 7.7 59.5 ± 9.5	31/6 42/12	NA NA	34/3/0 45/9/0	26/11 36/18	32.5 (32.0 ± 9.5) 47.6 (41.5 ± 18.0)
Iezzi *et al.*, 2015 (Italy)	Non-RCT	TACE + RFA TACE	40 20	68.2 ± 6.1 70.5 ± 5.8	24/16 16/4	4.7 ± 1.1 4.2 ± 1.7	24/16/0 3/17/0	NA NA	24 ± 8 24 ± 17
Liu *er al.*, 2014 (China)	Non-RCT	TACE + RFA TACE	45 43	45–75 44–78	36/9 43/34	4–15 5–14	13/20/12 10/23/10	30/15 30/13	NA NA
Othman *et al.*, 2014 (Egypt)	Non-RCT	TACE + RFA RFA TACE	20 20 20	49 (45–70) 49 (45–70) 49 (45–70)	40/20	5–7 5–7 5–7	NA NA NA	NA NA NA	NA NA NA
Song *et al.*, 2016 (Korea)	Non-RCT	TACE + RFA RFA TACE	87 43 71	60.4 (29–78) 62.0 (35–88) 60.0 (23–87)	70/17 31/12 53/18	2.5 (1.0–4.6) 2.2 (1.3–4.7) 2.5 (1.0–4.7)	80/7/0 37/6/0 68/3/0	62/23 35/8 41/30	33.3 (3.8–80.9) 33.3 (3.8–80.9) 33.3 (3.8–80.9)
Tang *et al.*, 2016 (China)	Non-RCT	TACE + RFA RFA TACE	40 49 43	48.3 ± 13.5 47.1 ± 13.3 45.8 ± 15.1	29/11 34/15 33/10	NA NA NA	18/22/0 22/27/0 19/24/0	14/26 15/34 13/30	NA NA NA
Yang *et al.*, 2008 (China)	RCT	TACE + RFA RFA TACE	24 12 11	59 ± 11.1 61.0 ± 10.4 57.6 ± 11.8	18/6 8/4 8/4	6.6 ± 0.6 5.2 ± 0.4 6.4 ± 1.0	11/5/1 8/6/1 10/5/0	5/34 8/18 7/1	NA NA NA
Yang *et al.*, 2009 (China)	Non-RCT	TACE + RFA RFA TACE	31 37 35	57.8 (43–78) 58.3 (38–80) 51.2 (30–74)	24/7 27/10 30/5	3.5 (1.7–7.3) 3.8 (2–6.4) 3.6 (1.2–8.0)	20/10/1 23/13/1 21/13/1	15/16 17/20 14/21	NA NA NA
Yin *et al.*, 2014 (China)	Non-RCT	TACE + RFA TACE	55 156	NA NA	47/8 138/18	5.9 (5–8) 6.0 (5–8)	48/7/0 136/20/0	35/20 115/41	23 (2–71) 23 (2–71)

NA, not applicable; Non-RCT, non-randomized control trial (retrospective or prospective cohort studies); RCT, randomized controlled trial; TACE, transcatheter arterial chemoembolization; RFA, radiofrequency ablation.

### Meta-analysis

There were 6 endpoints adopted to evaluate short- and long-term outcomes, including tumor response rates, recurrence-free survival, 1-year overall survival, 3-year overall survival, 5-year overall survival and postoperative complications. Dichotomous data was calculated using odds ratio (OR) with 95% confidence intervals (CI), and continuous data was calculated using mean difference (MD) with 95% CI.

### Tumor response

**Figure 4:**
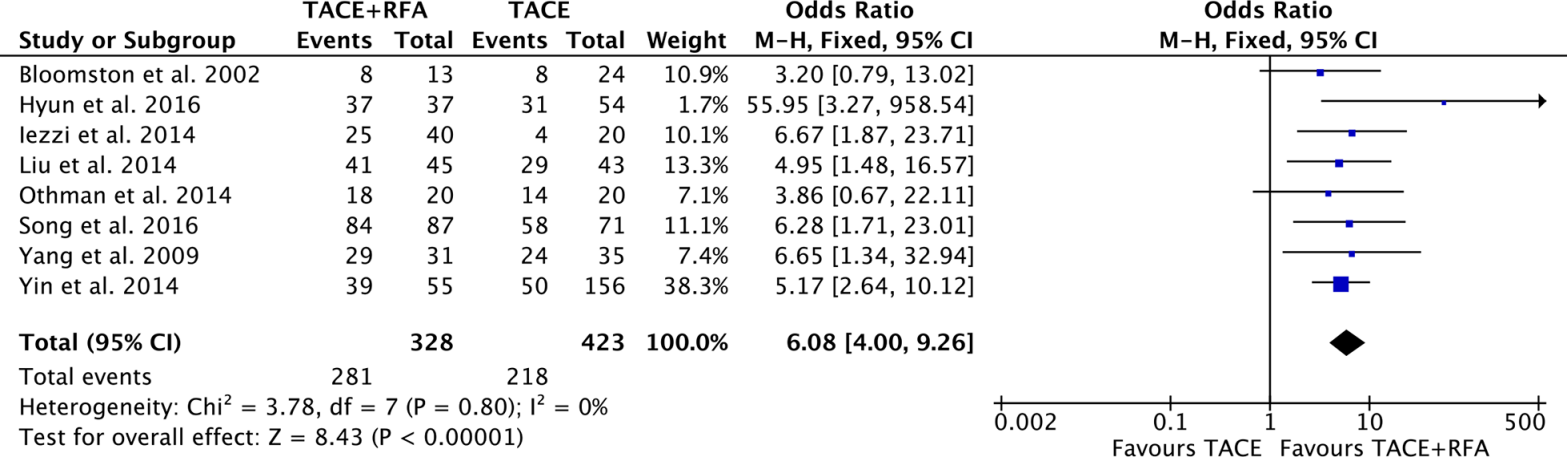
Tumor response rate of comparison TACE plus RFA with TACE alone intermediate stage hepatocellular carcinoma

Patients with complete response and partial response were both calculated for tumor response rates in this meta-analysis. Eight studies [16–18, 20–24] reported the tumor response outcome measurement. Heterogeneity was none among the studies (*P* = 0.80, *I^2^* = 0%), so the fixed effect model was used to pool the outcomes. The result (OR = 6.08, 95% CI = 4.00 to 9.26, *P* < 0.00001) indicated that tumor response rate of TACE plus RFA group is higher than that of TACE alone group (Figure 4).

### 1-year overall survival

**Figure 5:**
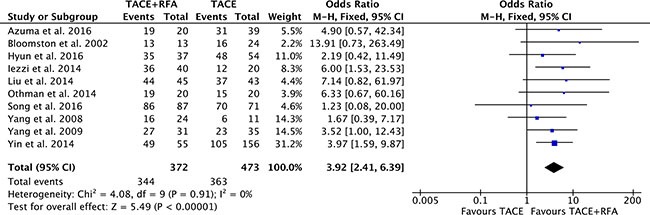
1-year overall survival rate of comparison TACE plus RFA with TACE alone intermediate stage hepatocellular carcinoma

Ten studies [15–23] (involving 845 participants) compared 1-year overall survival rates of TACE plus RFA group with TACE alone group. Heterogeneity was none among the studies (*P* = 0.91, *I^2^* = 0%), so the fixed effect model was used to pool the outcomes. The pool results showed that 1-year overall survival rates of TACE plus RFA group was higher (OR = 3.92, 95% CI = 2.41 to 6.39, *P* < 0.00001) than that of TACE alone group (Figure 5).

### 3-year overall survival

**Figure 6:**
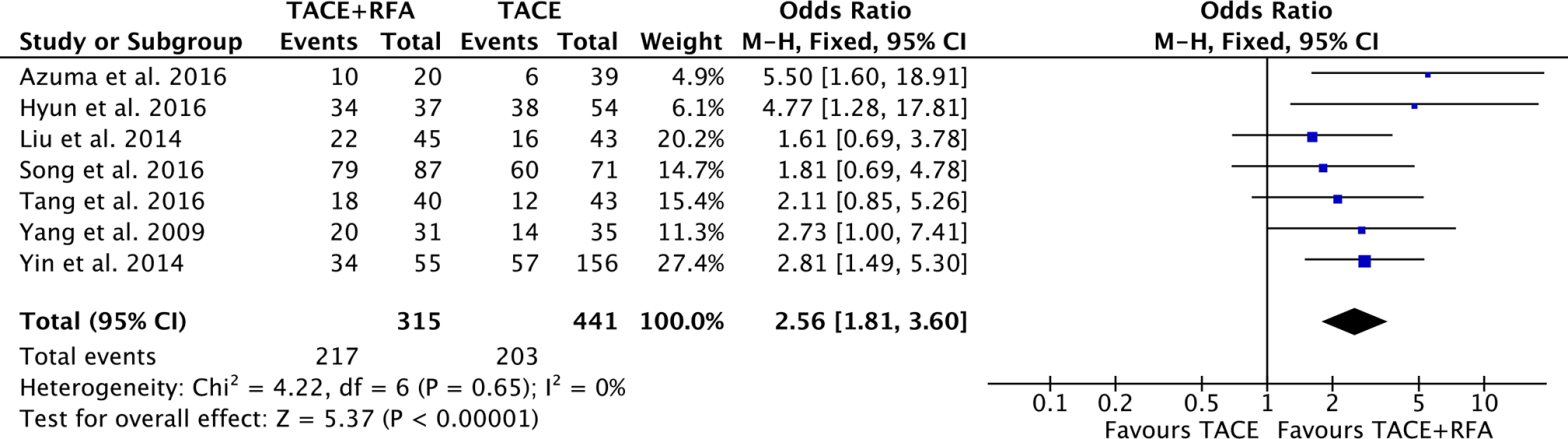
3-year overall survival rate of comparison TACE plus RFA with TACE alone intermediate stage hepatocellular carcinoma

There were seven studies [16, 18–20, 23–25] compared 3-year overall survival rates of TACE plus RFA group with TACE alone group. Heterogeneity was none among the studies (*P* = 0.65, *I^2^* = 0%), so the fixed effect model was used to pool the outcomes. The pool results showed that 3-year overall survival rates of TACE plus RFA group was higher (OR = 2.56, 95% CI = 1.81 to 3.60, *P* < 0.00001) than that of TACE alone group (Figure 6).

### 5-year overall survival

**Figure 7:**
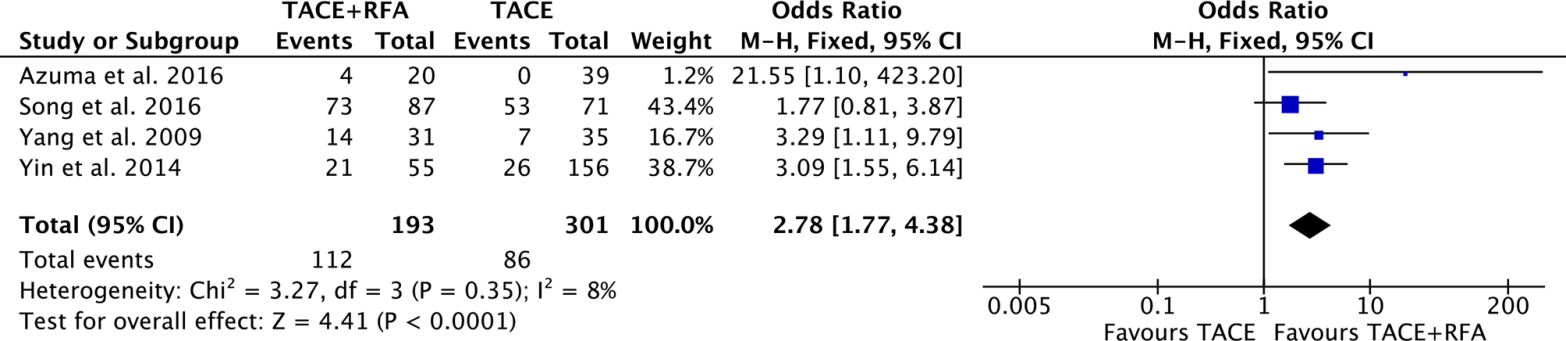
5-year overall survival rate of comparison TACE plus RFA with TACE alone intermediate stage hepatocellular carcinoma

Four studies [16, 19, 20, 23] with 494 patients compared 5-year overall survival rates of TACE plus RFA group with TACE alone group. Heterogeneity was low among the studies (*P* = 0.35, *I^2^* = 8%), so the fixed effect model was used to pool the outcomes. The pool results showed that 5-year overall survival rates of TACE plus RFA group was higher (OR = 2.78, 95% CI = 1.77 to 4.38, *P* < 0.0001) than that of TACE alone group (Figure 7).

### Recurrence-free survival

**Figure 8:**
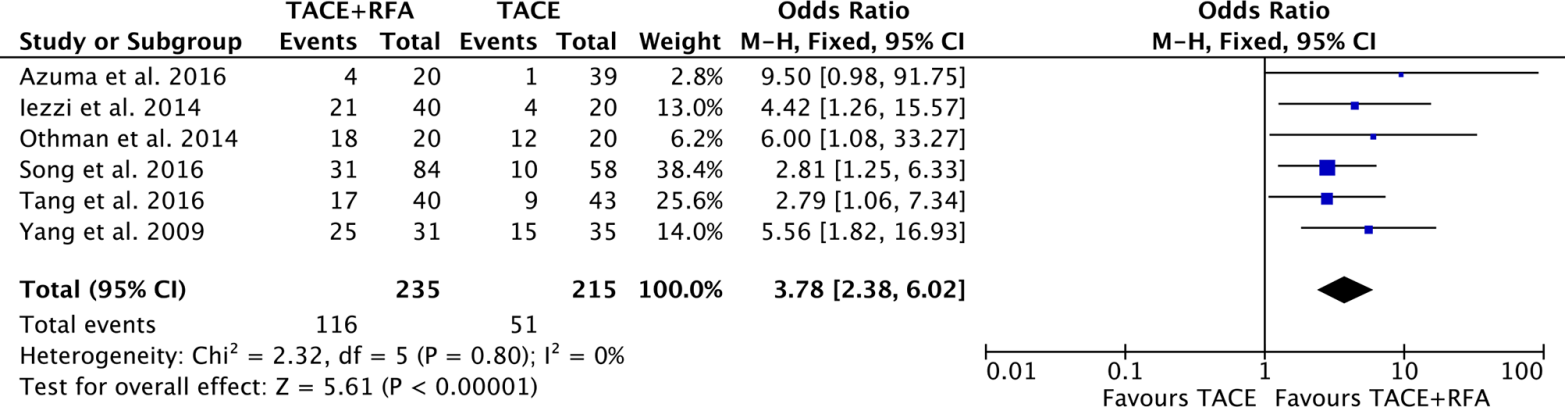
Recurrence-free survival rate of comparison TACE plus RFA with TACE alone intermediate stage hepatocellular carcinoma

There were six studies [16, 17, 19, 20, 22, 25] reported the recurrence-free survival rates. Heterogeneity was none among the studies (*P* = 0.80, *I^2^* = 0%), so the fixed effect model was used to pool the outcomes. Recurrence-free survival rates of TACE plus RFA group was significantly higher than that of TACE alone group (OR = 3.78, 95% CI: 2.38 to 6.02, *P* < 0.00001) (Figure 8).

### Postoperative complications

**Figure 9:**
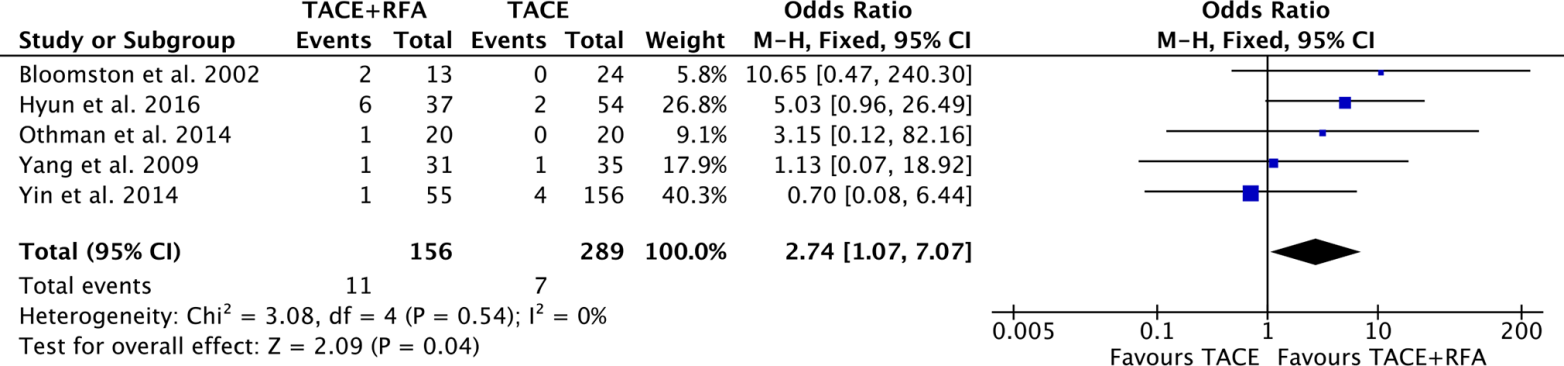
Postoperative complications of comparison TACE plus RFA with TACE alone intermediate stage hepatocellular carcinoma

There were five studies [17, 18, 20, 21, 23] reported moderate and major postoperative complications. Heterogeneity was none among the studies (*P* = 0.54, *I^2^* = 0%), so the fixed effect model was used to pool the outcomes. The pool results showed that postoperative complications of TACE plus RFA group were higher (OR = 2.74, 95% CI = 1.07 to 7.07, *P* = 0.04) than that of TACE alone group (Figure 9).

### Publication bias

**Figure 10:**
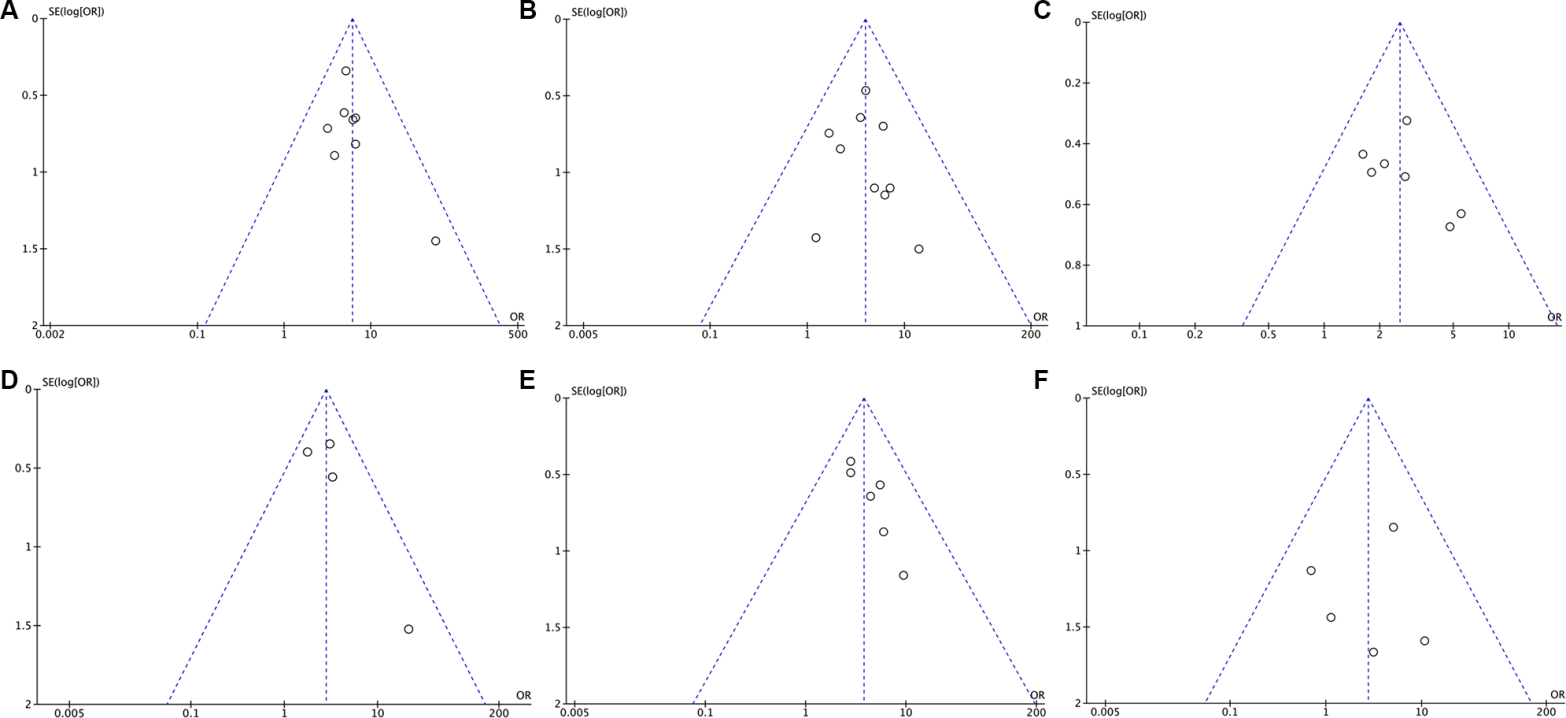
The funnel plots of TACE plus RFA versus TACE alone for intermediate stage hepatocellular carcinoma **(A)** Tumor response rate; **(B)** 1-year overall survival rate; **(C)** 3-year overall survival rate; **(D)** 5-year overall survival rate; **(E)** Recurrence-free survival rate; **(F)** Postoperative complications.

The symmetry the funnel plot was used to assess the reliability of publication bias in this meta-analysis [26]. The shape of six funnel plots was bilateral symmetry and basically inverted. So, these results showed that all comparisons in this meta-analysis had no publication bias (Figure 10).

## DISCUSSION

Hepatocellular carcinoma has high malignant degree that usually occurs to associate with cirrhosis [2, 27]. The median survival of hepatocellular carcinoma is approximately 6 to 20 months, because many patients with chronic liver disease are diagnosed in the late stages [2]. When surgical resection or liver transplantation is not a suitable option, RFA and TACE have been established as valuable treatment modalities for patients with hepatocellular carcinoma [5, 6]. For unresectable hepatocellular carcinoma, RFA is a reasonable approach [28, 29]. RFA is especially appropriate for patients with a single carcinoma less than 4 cm in diameter, and can attain the best outcome [30]. But, RFA alone treatment for hepatocellular carcinoma should not be applied in larger than 5 cm, because it is hardly to obtain complete necrosis and satisfactory local tumor control in large carcinoma [14]. Therefore, small hepatocellular carcinoma with treated by RFA is less local recurrence and attains better prognosis [31]. TACE is used most often in the treatment for large unresectable hepatocellular carcinoma that is not applicable to other therapies such as surgical resection or RFA [32]. And yet, all feeding arteries of carcinoma are hardly to chemoembolization by TACE, because they have multiple sources and can develop newly collateral arteries [33]. Local recurrences are the majority of recurrences after treated by TACE, thus an effective adjuvant therapy is needed to prevent or delay recurrence [34].

When TACE is used in combination with RFA for intermediate stage hepatocellular carcinoma, it can theoretically overcome the limitations of TACE or RFA using alone [35, 36]. There are two main benefits to the combination approach. Firstly, tumor burden can be reduced by chemoembolization of TACE, and the ablation rate by RFA would be increased [37]. Secondly, the range of carcinoma and undetected satellite nodules are labeled after TACE is initially performed. Thus, it provides guidance and increases the chances of complete ablation of both main tumor and satellite nodules by RFA [38]. The use of TACE plus RFA of treatment for small hepatocellular carcinoma is a common practice [39]. However, it is still controversial whether the effect of TACE plus RFA for intermediate stage hepatocellular carcinoma is better than that of TACE alone [13, 14]. To our knowledge, a meta-analysis will be helpful to attain definitive proof to solve those clinically controversial problems. Thus, we performed this meta-analysis to help stratify the effects of this combination approach for intermediate stage hepatocellular carcinoma.

In the present meta-analysis, tumor sizes that were reported in 11 included studies were from 0.4 to 8 cm and were no obviously different between two groups. The pool results showed that TACE plus RFA group attained higher tumor response rates than TACE alone. Additionally, recurrence-free survival rates and 1-, 3- and 5-year overall survival rates of TACE plus RFA group were higher than that of TACE alone group. Although postoperative complications of TACE plus RFA group was higher than that of TACE alone group, most complications in all studies are very mild and serious complications including tumor seeding, liver failure, or treatment-related death were not observed. So, this study revealed that the use of TACE plus RFA for intermediate stage hepatocellular carcinoma can improve higher tumor response rates and survival rates than TACE alone.

This meta-analysis has several limitations. First, this meta-analysis includes both randomized controlled trials and retrospective studies, but there is only one randomized controlled trial. So, it was relatively low quality of the evidence for all pooled results. Second, there are a total of 11 studies with 928 participators, but the sample sizes 5 of them are small. It cannot apply further sensitivity analysis on the factors affecting outcomes. Therefore, some well-designed, large, and multi-center randomized controlled trials are desperately needed to obtain further evidence.

In conclusion, the use of TACE plus RFA for intermediate stage hepatocellular carcinoma can attain higher tumor response rates and improve survival rates than TACE alone. Patient with intermediate stage hepatocellular carcinoma treated by this combine approach is effective and safe.

## MATERIALS AND METHODS

### Study inclusion and exclusion criteria

The inclusion criteria of this meta-analysis were shown as follow: (1) comparison TACE plus RFA with TACE alone for intermediate stage hepatocellular carcinoma; (2) randomized controlled trials or retrospective studies; (3) patients of two groups with average basic clinical characters; (4) studies with outcomes information of tumor response rates, postoperative complications recurrence-free survival rates, and overall survival rates.

The exclusion criteria of this meta-analysis were list as follow: (1) studies without original data, such as abstracts, case reports, expert opinions, editorials, reviews and letters; (2) studies with a sample size less than 30.

### Information sources and search strategy

A systematic electronic databases search of MEDLINE, EMBASE and CENTRAL was separately performed by two reviewers to identify all relevant studies available until October 31th 2016. When a study was uncertain, we consulted the corresponding author for more information. Ongoing clinical trials were searched from two websites of trial registries (www.clinicaltrials.gov, www.clinicaltrialsregister.eu).

The search strategy followed the identification and screening guidelines established by the Preferred Reporting Items for Systematic Reviews and Meta-Analyses (PRISMA) statement. The subject headings (MeSH) search and text word search were used, including “hepatocellular carcinoma”, “transcatheter arterial chemoembolization” and “radiofrequency ablation”. These terms were used in different combinations. The included study was restricted to human beings and English language. A manual research was performed by searching all references of all identified studies. This research progress was repeated over and again to ensure all related studies could be identified. The research was independently completed by two reviewers (D-J. Yang and K-L. Luo).

### Data collection

### Studies selection

The flow diagram of Figure 1 showed the detailed of studies selection process. Data of the included studies were independently extracted and evaluated by two review authors (H. Liu and B. Cai). Studies were identified by the relevance of titles and abstracts and classified as duplicate, excluded, included, or uncertain according to exclusion and inclusion criteria. All disagreements were resolved by consensus.

### Data extraction and management

Data were independently extracted from each study by two reviewers (H. Liu and B. Cai), including first author, publication year, country, patient characteristics, demographics, study design, sample size, Child-Pugh score, follow up, complications and survival. Data accuracy and completeness were checked by two other authors (G-Q. Tao and X-J. Hou). All discrepancies were resolved by consensus.

### Risk of bias assessment

Risk of bias was assessed using the criteria of Cochrane Collaboration's tool by two reviewers (X-F. Su and F. Ye) [26]. Good quality criteria studies were as follow: sequence generation randomized; allocation concealment; blinding every participant; complete outcome data; and non-selective outcome reporting. The funnel plots were used to analyze publication bias. All discrepancies were resolved by consensus.

### Statistical analysis and synthesis

Review Manager (version 5.3) was used to perform the statistical analysis. Dichotomous data was calculated using OR with 95% CI, and continuous data was calculated using MD with 95% CI. If the *P* < 0.05 and 95% CI did not include the value 1, OR was considered statistically significant.

Cochran's *Q* test and by the degree of inconsistency (*I^2^*) were used to assess heterogeneity among pool results. Either fixed-effect model or random-effect model were used adjust for possible heterogeneity. If *P* < 0.05 and *I^2^* < 50%, the fixed-effect model was used to pool data. Otherwise, the random-effect model was used. *P* < 0.05 was considered as statistical significance in the integration results.

This study was also supported by Science and Technology Project Foundation of Nanjing (grant no: 201303032); Medical Science and Technology Development Foundation of Jiangsu University (grant no: JLY20140160).
